# Insoluble dietary fibre intake is associated with lower prevalence of newly-diagnosed non-alcoholic fatty liver disease in Chinese men: a large population-based cross-sectional study

**DOI:** 10.1186/s12986-019-0420-1

**Published:** 2020-01-13

**Authors:** Yang Xia, Shunming Zhang, Qing Zhang, Li Liu, Ge Meng, Hongmei Wu, Xue Bao, Yeqing Gu, Shaomei Sun, Xing Wang, Ming Zhou, Qiyu Jia, Kun Song, Qijun Wu, Kaijun Niu, Yuhong Zhao

**Affiliations:** 1 Department of Clinical Epidemiology, Shengjing Hospital of China Medical University, No. 36, San Hao Street, Shenyang, 110004 Liaoning China; 2 Nutritional Epidemiology Institute and School of Public Health, Tianjin Medical University, 22 Qixiangtai Road, Heping District, Tianjin, 300070 China; 3 Health Management Centre, Tianjin Medical University General Hospital, Tianjin, China

**Keywords:** Non-alcoholic fatty liver disease, Prevalence, Dietary fibre, OR

## Abstract

**Background:**

The health benefits of dietary fibre (DF) intake on non-alcoholic fatty liver disease (NAFLD) are controversial. Thus, this large cross-sectional study aimed to determine the associations between DF intake and the prevalence of newly-diagnosed NAFLD in a large general population.

**Methods:**

A total of 23,529 participants were enrolled in the analyses. Newly-diagnosed NAFLD was diagnosed according to liver ultrasonography and history of alcohol intake**.** DF intake was assessed using a validated self-administered food frequency questionnaire. Logistic regression analysis was applied to estimate the associations between DF intake and NAFLD.

**Results:**

In total, 18.81% (*n* = 4426) of the participants were newly diagnosed with NAFLD. Compared with the participants in the lowest quartile of DF intake, the ORs (95% CI) for the participants in the highest quartile were 0.81 (0.67, 0.97), 0.78 (0.62, 0.99), and 0.85 (0.62, 1.17) for all participants, men, and women, respectively. Compared with the participants in the lowest quartile of insoluble DF intake, the ORs (95% CI) for the participants in the highest quartile were 0.70 (0.58, 0.85), 0.60 (0.47, 0.76), and 0.95 (0.68, 1.32) in all participants, men, and women, respectively. No association was observed between soluble DF intake and NAFLD. DF from whole grain, soy foods, vegetables, and nuts but not fruits were negatively associated with NAFLD.

**Conclusions:**

A higher intake of insoluble DF is associated with lower prevalence of newly-diagnosed NAFLD in men. Moreover, intake DF from whole grain, soy foods, vegetables, and nuts, but not fruits have favorable effect on the prevalence of newly diagnosed NAFLD. Further cohort studies and randomized controlled trials are needed to validate this finding.

## Introduction

Non-alcoholic fatty liver disease (NAFLD) is a liver disease associated with obesity, insulin resistance, type 2 diabetes mellitus, hypertension, hyperlipidaemia, and metabolic syndrome [1]. NAFLD has a potentially progressive course that leads to liver fibrosis, cirrhosis, hepatocellular carcinoma and liver transplantation [2]. In 2018, approximately 25% of the global population has NAFLD [3]. Urbanisation of many Asian countries in the past 2 decades has led to the prevalence of sedentary lifestyles and overnutrition, setting the stage for the epidemic of obesity and consequently NAFLD [2]. We previously found that NAFLD is prevalent in 30.7% of Chinese adults [4]. Considering the disease burden and high prevalence of NAFLD, it is important to identify modifiable risk factors and develop preventive strategies. However, medications for treating NAFLD lack efficacy and safety, and lifestyle management, including sustained weight loss, health dietary, and increased physical activity, remains an important approach in treating NAFLD [5].

Previous studies found that consumption of dietary components is associated with the prevalence of NAFLD [4, 6, 7]. One study reported lower intakes of dietary fibre (DF) and higher intakes of carbohydrates, saturated fats, cholesterol, fructose, and animal protein, as well as greater n-6:n-3 ratios in the NAFLD population [8]. DF is a kind of non-starch polysaccharide carbohydrate which usually includes cellulose, insoluble hemicelluloses, and lignin [9]. A case-control study, of 25 non-alcoholic steatohepatitis (NASH) patients and 25 matched healthy controls found that the mean daily DF intake was almost 50% lower in the NASH patients versus controls without adjusting for confounding factors [10]. Subsequent studies also demonstrated that DF intake is inversely associated with NAFLD and the risk factors of NAFLD [11–13]. However, these studies are limited by small sample size, incomplete adjustment, or use NASH and hepatic fat fraction as endpoints rather than NAFLD. A recent cross-sectional study of 3882 participants found that there is no consistent association between DF intake and NAFLD [14]. However, the analyses were conducted in an aging population, which would limit the generalisation of their findings.

Thus, this study aimed to explore the associations between DF consumption and the prevalence of newly diagnosed NAFLD in a large general adult population in China.

## Materials and methods

### Study design and population

This cross-sectional study is based on the Tianjin Chronic Low-grade Systemic Inflammation and Health Cohort Study, which is a large prospective dynamic cohort study focusing on the relationships between chronic low-grade inflammation and the health status of a general population living in Tianjin, China. The design and data collection of this cohort study has been described in detail previously [15]. The present study has been approved by the Institutional Review Board of the Tianjin Medical University and has been performed in accordance with the ethical standards laid down in the 1964 Declaration of Helsinki and its later amendments. All participants gave written informed consent prior to study inclusion.

In total, 32,165 participants who underwent abdominal ultrasound and completed a study questionnaire reporting personal information, dietary intake, lifestyles and health condition between May 2013 to December 2016 were included. We excluded participants who had a history of CVD (cardiovascular disease) (*n* = 1579) or cancer (*n* = 254), or those with missing data (*n* = 1130). In addition, we excluded participants who had a history of NAFLD (*n* = 4437), alcoholic fatty liver disease (*n* = 1110), chronic hepatitis B or C, autoimmune liver disease, and those who had previous liver surgery (*n* = 126). Finally, 23,529 participants were included in the analysis.

### Assessment of dietary data

Dietary intake was assessed using a modified version of the food frequency questionnaire (FFQ) that included 100 food items (the initial version of the FFQ included 81 food items [4]) with specified serving sizes one time. The FFQ included seven frequency categories ranging from ‘almost never’ to ‘twice or more per day’ for foods and eight frequency categories ranging from ‘almost never’ to ‘four or more times per day’ for beverages in the last month. The reproducibility and validity of the questionnaire were assessed in a random sample of 150 participants from our cohort using data from repeated measurements of the FFQ approximately 3 months apart and 4-d weighed diet records (WDR). The Spearman rank correlation coefficient for energy intake between two FFQs administered 3 months apart was 0.68 (*P* <  0.05). The correlation coefficients for food items (i.e. fruits, vegetables, fish, meat and beverages) between two FFQs administered 3 months apart ranged from 0.62 to 0.79 (all *P* <  0.05). Meanwhile, the Spearman’s rank correlation coefficient for energy intake by the WDR and the FFQ was 0.49 (*P* < 0.05). Correlation coefficients for nutrients (i.e. vitamin C, vitamin E, PUFA, SFA, carbohydrate and Ca) using the WDR and the FFQ ranged from 0.35 to 0.54 (DF, 0.44; insoluble DF, 0.49; soluble DF, 0.42) (all *P* < 0.05).

The mean daily intake of nutrients, including DF, was calculated by using an ad hoc computer program developed to analyses the questionnaire. Consumption of food items was calculated by multiplying the portion size (g/time) by the frequency of each food item consumed per day. Furthermore, the Chinese food composition tables [16] were used as the nutrient database to calculate the intake of nutrients. Nutrients intake was calculated by first multiplying the gram of consumption for each food item by its nutrient content per gram and then adding the nutrient contributions across all food items.

### Assessment of newly-diagnosed NAFLD

Liver ultrasonography was conducted by trained sonographers using a TOSHIBA SSA-660A ultrasound machine (Toshiba, Tokyo, Japan), with a 2–5-MHz curved array probe. Drinking habit was assessed according to the revised definition and treatment guidelines for NAFLD by the Chinese Association for the Study of Liver Disease in 2010 [17], with ‘heavy drinking’ defined as > 140 g alcohol intake per week in men and > 70 g per week in women. Total alcohol intake in the past week was assessed by using the FFQ. Participants were diagnosed as having NAFLD using abdominal ultrasonography (brightness of liver and a diffusely echogenic change in the liver parenchyma) and no history of heavy drinking. Participants with a self-reported history of or were previously diagnosed with NAFLD were excluded in the present study. Thus, all participants with NAFLD in the present study were newly diagnosed.

### Assessment and definition of other variables

Information on the sociodemographic variables, including sex, age, educational level, employment, smoking status, drinking status, and household income, was collected using a questionnaire. Physical activity (PA) in the most recent week was assessed using the short form of the International Physical Activity Questionnaire [18]. The questionnaire asked whether subjects had performed any activities from the following categories during the previous week: walking; moderate activity (household activity or child care); and vigorous activity (running, swimming, or other sports activities). Metabolic equivalent (MET) hours per week were calculated using corresponding MET coefficients (3.3, 4.0 and 8.0, respectively) according to the following formula: MET coefficient of activity × duration (hours) × frequency (days). Total PA levels were assessed by combining the scores for different activities.

Body mass index (BMI) was calculated as weight in kilograms divided by the square of height in meters (kg/m^2^). Blood pressure (BP) was measured twice from the upper left arm using a TM-2655P automatic device (A&D CO., Tokyo, Japan) after 5 min of rest in a seated position. The mean of these 2 measurements was taken as the BP value. Hypertension was defined as average systolic BP ≥ 140 mmHg or average diastolic BP ≥ 90 mmHg or use of antihypertension medications [19]. Fasting blood samples were collected via venipuncture of the cubital vein and immediately mixed with EDTA. Fasting blood glucose (FBG) and lipids were collected in siliconised vacuum plastic tubes. FBG was measured using the glucose oxidase method, triglycerides (TG) and total cholesterol (TC) were measured using enzymatic methods, and high-density lipoprotein cholesterol was measured via the chemical precipitation method using reagents from Roche Diagnostics on an automatic biochemistry analyzer (Roche Cobas 8000 modular analyzer, Mannheim, Germany). HbA1c separation and quantification were conducted using a high-performance liquid chromatography analyzer (HLC-723 G8; Tosoh, Tokyo, Japan). In addition, an oral glucose tolerance test was performed, and postprandial glucose was determined in blood samples obtained 2 h after oral administration of a standard 75 g glucose solution. Type 2 diabetes was defined according to the 2014 American Diabetes Association criteria as fasting blood glucose ≥7.0 mmol/, or 2-h postprandial blood glucose ≥11.1 mmol/l, or HbA1c ≥ 6.5% (48 mmol/mol), or a history of diabetes [20]. Hyperlipidaemia was defined as TC ≥ 5.20 mmol/L, or TG ≥ 1.70 mmol/L, or self-reported clinically diagnosed hyperlipidaemia according to 2016 Chinese guidelines for the management of dyslipidaemia in adults [21].

### Statistical analysis

Population characteristics were described according to the quartiles of dietary fiber intake in men and women. Continuous variables were presented as least-square means and 95% confidence interval (CI). Categorical variables were presented as percentage. Analysis of variance or logistic regression test were used to evaluate differences in continuous variables and categorical variables, between participants with and without NAFLD. Quartiles were categorised across the intake of DF based on the distribution of the intake of DF by sex. The quartile 1 to 4 in total participants were combined from the same quartiles in women and men. Associations between quartile categories of DF intake and NAFLD status were examined using logistical regression analysis. Newly-diagnosed NAFLD status was used as a dependent variable, and quartile categories of DF intake were used as independent variables. Odds ratio (OR) and 95% CI were calculated. The linear trend cross increasing quartiles was tested using the median value of each quartile as a continuous variable based on logistic regression. The crude model was used to calculate the crude OR without any adjustment. Model 1 adjusted for age, sex, and BMI. Model 2 was additionally adjusted for type 2 diabetes (yes/no), hypertension (yes/no), hyperlipidaemia (yes/no), physical activity (Mets × hours/week), educational level (≥ college graduate or not), household income (≥ 10,000 yuan per month or not), smoking status (current smoker, ex-smoker, or non-smoker), drinking status (drinking every day, drinking sometimes, ex-drinker, or non-drinker), employment status (managers, professionals, or other), energy intake (kcal/d), total carbohydrate intake (g/d), total fat intake (g/d), sweet foods intake (g/d), red meat intake (g/d), white meat intake (g/d), DHA + EPA intake (g/d), and family history of CVD (yes/no), hypertension (yes/no), and diabetes (yes/no) based on model 1. Multivariate logistic regression collinearity diagnosis analysis was performed for adjustment models and no collinearity between variables was found. All analyses were performed using the Statistical Analysis System 9.3 edition for Windows (SAS Institute Inc.). All *P* values were two-tailed and the difference was defined to be significant when *P* < 0.05.

## Results

### Participant characteristics

Among the 23,529 participants who were eligible for the final analyses, 4426 (18.81%) had newly-diagnosed NAFLD. The socio-demographic, behavioural, anthropometric, dietary, and clinical characteristics according to the quartiles of DF intake in men and women are shown in Table 1. In men, participants who had higher intake of DF tended to be non-smoker, non-drinker, and managers, who had higher levels of BMI, physical activity, and educational status, but lower chance to have hyperlipidaemia and family history of CVD. In women, participants who had higher intake of DF tended to be older, non-smoker, and non-drinker, who had higher level of alanine aminotransferase, aspartate aminotransferase, physical activity, but lower change to be managers, low proportion to have type 2 diabetes, and family history of CVD and diabetes. Moreover, both in men and women, participants who had higher intake of DF tended to had higher intake of total energy, DF, soluble DF, insoluble DF, carbohydrate, fat, whole grain, soy foods, vegetables, fruits, nuts, sweet foods, red meat, white meat, and docosahexaenoic acid + eicosapentaenoic acid (all *P* < 0.05).

**Table 1:** Participant characteristics according to fibre intake^a^

	Categories of dietary fibre intake				*P* for trend ^b^
Men (*n* = 10,998)	Level 1 (*n* = 2750)	Level 2 (*n* = 2749)	Level 3 (*n* = 2749)	Level 4 (*n* = 2750)	
Intake of dietary fibre (range, g/d)	0.839, 17.417	17.420, 24.134	24.135, 33.692	33.697, 173.414	
NAFLD (%)	30.1	28.3	28.0	28.5	0.31
Age (years)	39.5 (39.0, 39.9) ^c^	39.2 (38.7, 39.6)	39.4 (39.0, 39.8)	39.3 (38.9, 39.7)	0.80
BMI	24.9 (24.8, 25.0)	24.8 (24.7, 25.0)	25.1 (24.9, 25.2)	25.1 (24.9, 25.2)	0.02
ALT (U/L)	23.2 (22.7, 23.6)	22.8 (22.3, 23.2)	23.0 (22.6, 23.5)	22.7 (22.3, 23.2)	0.26
AST (U/L)	20.0 (19.5, 20.4)	20.0 (19.6, 20.4)	20.1 (19.6, 20.5)	20.4 (20.0, 20.9)	0.14
Type 2 diabetes (%)	4.4	3.6	3.8	3.8	0.36
Hypertension (%)	26.7	26.7	26.5	25.9	0.47
Hyperlipidaemia (%)	49.2	45.9	46.6	44.9	< 0.01
Physical activity (Mets × hours/week)	9.3 (8.8, 9.7)	11.1 (10.5, 11.6)	13.2 (12.6, 13.9)	14.3 (13.6, 15.0)	< 0.0001
Energy intake (kcal/d)	1666.3 (1652.1, 1680.5)	2103.2 (2085.3, 2121.2)	2247.8 (2228.7, 2267.1)	2346.3 (2326.4, 2366.4)	< 0.0001
Total fibre intake (g/d)	12.8 (12.7, 12.9)	20.6 (20.5, 20.8)	28.3 (28.1, 28.5)	46.4 (46.0, 46.7)	< 0.0001
Soluble fibre intake (g/d)	5.3 (5.2, 5.4)	9.1 (9.0, 9.2)	13.2 (13.1, 13.4)	24.2 (24.0, 24.5)	< 0.0001
Insoluble fibre intake (g/d)	7.4 (7.3, 7.5)	11.2 (11.1, 11.3)	14.5 (14.4, 14.7)	21.0 (20.8, 21.2)	< 0.0001
Total carbohydrate intake (g/d)	259.2 (256.0, 262.3)	345.3 (341.1, 349.5)	411.5 (406.5, 416.6)	541.2 (534.6, 547.9)	< 0.0001
Total fat intake (g/d)	35.2 (34.7, 35.7)	46.0 (45.3, 46.6)	54.4 (53.6, 55.2)	69.6 (68.6, 70.6)	< 0.0001
Whole grain intake (g/d)	6.8 (6.5, 7.1)	11.4 (10.9, 11.9)	14.8 (14.2, 15.4)	19.0 (18.3, 19.9)	< 0.0001
Soy foods intake (g/d)	12.7 (12.2, 13.1)	23.0 (22.2, 23.9)	28.5 (27.5, 29.6)	34.2 (32.9, 35.5)	< 0.0001
Vegetables intake (g/d)	162.2 (159.6, 164.9)	239.4 (235.5, 243.3)	296.6 (291.8, 301.4)	389.4 (383.1, 395.8)	< 0.0001
Fruits intake (g/d)	119.4 (116.6, 122.2)	227.5 (222.2, 232.9)	335.6 (327.8, 343.5)	607.7 (593.7, 622.1)	< 0.0001
Nuts intake (g/d)	3.4 (3.2, 3.5)	5.5 (5.3, 5.7)	7.2 (6.9, 7.4)	9.3 (8.9, 9.6)	< 0.0001
Sweet foods intake (g/d)	8.3 (7.8, 8.7)	14.3 (13.5, 15.1)	18.0 (17.0, 19.1)	29.5 (27.9, 31.3)	< 0.0001
Red meat intake (g/d)	26.3 (25.3, 27.4)	32.5 (31.3, 33.8)	34.6 (33.3, 36.0)	34.1 (32.8, 35.5)	< 0.0001
White meat intake (g/d)	16.6 (15.9, 17.4)	21.0 (20.1, 21.9)	23.9 (22.9, 24.9)	26.6 (25.5, 27.7)	< 0.0001
DHA + EPA intake (g/d)	3.2 (3.1, 3.3)	4.0 (3.9, 4.1)	4.5 (4.4, 4.6)	5.6 (5.5, 5.7)	< 0.0001
Education (≥ College graduate, %)	64.6	69.9	70.1	69.0	< 0.01
Household income (≥ 10,000 Yuan, %)	32.1	37.9	37.0	35.1	0.17
Smoking status (%)					
Smoker	39.7	35.8	34.0	32.4	< 0.0001
Ex-smoker	9.3	8.8	9.4	8.3	0.28
Non-smoker	51.0	55.4	56.7	59.3	< 0.0001
Drinker (%)					
Everyday	7.0	6.7	5.6	6.0	0.07
Sometime	71.3	72.9	73.4	70.3	0.31
Ex-drinker	11.0	9.6	11.3	10.7	0.73
Non-drinker	10.8	10.8	9.8	13.0	0.01
Employment status (%)					
Managers	39.3	42.1	43.4	44.9	< 0.0001
Professionals	21.9	21.7	21.3	20.5	0.20
Other	38.9	36.2	35.3	34.6	< 0.01
Family history of diseases (%)					
CVD	25.0	25.5	25.8	22.3	0.02
Hypertension	45.1	44.9	47.3	43.2	0.33
Diabetes	22.6	22.2	22.3	21.0	0.18
Women (*n* = 12,531)	Level 1 (*n* = 3133)	Level 2 (*n* = 3133)	Level 3 (*n* = 3132)	Level 4 (*n* = 3133)	
Intake of dietary fibre (range, g/d)	0.400, 16.161	16.164, 22.284	22.287, 30.760	30.764, 193.433	
NAFLD (%)	10.0	9.8	10.4	10.2	0.89
Age (years)	38.0 (37.7, 38.4)	38.6 (38.2, 39.0)	39.1 (38.8, 39.5)	39.1 (38.7, 39.5)	< 0.01
BMI	22.5 (22.4, 22.6)	22.6 (22.4, 22.7)	22.6 (22.5, 22.7)	22.6 (22.5, 22.7)	0.07
ALT (U/L)	14.3 (14.1, 14.5)	14.4 (14.2, 14.6)	14.5 (14.3, 14.8)	14.6 (14.4, 14.8)	0.04
AST (U/L)	17.4 (17.1, 17.8)	17.8 (17.4, 18.1)	17.9 (17.5, 18.2)	18.3 (17.9, 18.7)	< 0.001
Type 2 diabetes (%)	2.1	1.7	1.5	1.3	0.01
Hypertension (%)	11.8	11.8	12.6	12.5	0.25
Hyperlipidaemia (%)	31.5	31.8	33.0	33.0	0.21
Physical activity (Mets × hours/week)	7.1 (6.8, 7.4)	9.1 (8.7, 9.5)	9.3 (8.9, 9.7)	10.2 (9.7, 10.7)	< 0.0001
Energy intake (kcal/d)	1456.3 (1443.7, 1469.0)	1906.6 (1890.1, 1923.2)	2134.7 (2116.2, 2153.3)	2304.6 (2284.7, 2324.7)	< 0.0001
Total fibre intake (g/d)	11.9 (11.8, 12.0)	19.1 (19.0, 19.3)	26.0 (25.8, 26.2)	42.8 (42.4, 43.1)	< 0.0001
Soluble fibre intake (g/d)	4.9 (4.8, 5.0)	8.3 (8.2, 8.4)	12.0 (11.8, 12.1)	21.8 (21.6, 22.0)	< 0.0001
Insoluble fibre intake (g/d)	6.8 (6.7, 6.9)	10.5 (10.4, 10.6)	13.5 (13.4, 13.6)	19.9 (19.7, 20.1)	< 0.0001
Total carbohydrate intake (g/d)	237.6 (234.9, 240.3)	316.5 (313.0, 320.2)	372.1 (367.9, 376.4)	493.1 (487.5, 498.7)	< 0.0001
Total fat intake (g/d)	29.2 (28.8, 29.6)	38.1 (37.6, 38.6)	44.5 (43.9, 45.1)	57.8 (57.1, 58.6)	< 0.0001
Whole grain intake (g/d)	6.6 (6.4, 6.9)	11.2 (10.8, 11.6)	13.8 (13.3, 14.3)	17.2 (16.6, 17.8)	< 0.0001
Soy foods intake (g/d)	11.8 (11.4, 12.2)	20.7 (20.0, 21.4)	26.3 (25.4, 27.2)	33.4 (32.3, 34.6)	< 0.0001
Vegetables intake (g/d)	149.2 (146.9, 151.6)	217.1 (213.7, 220.6)	271.0 (266.7, 275.3)	367.5 (361.7, 373.4)	< 0.0001
Fruits intake (g/d)	147.1 (144.3, 150.0)	255.4 (250.5, 260.4)	359.1 (352.1, 366.2)	626.2 (614.1, 638.6)	< 0.0001
Nuts intake (g/d)	3.2 (3.1, 3.3)	5.0 (4.9, 5.2)	6.5 (6.3, 6.7)	8.2 (7.9, 8.4)	< 0.0001
Sweet foods intake (g/d)	12.1 (11.6, 12.8)	17.8 (16.9, 18.7)	21.6 (20.5, 22.7)	30.4 (28.9, 32.0)	< 0.0001
Red meat intake (g/d)	15.9 (15.3, 16.6)	19.3 (18.5, 20.1)	20.2 (19.4, 21.0)	20.6 (19.8, 21.4)	< 0.0001
White meat intake (g/d)	12.1 (11.6, 12.6)	14.0 (13.5, 14.6)	16.0 (15.4, 16.7)	17.8 (17.0, 18.5)	< 0.0001
DHA + EPA intake (g/d)	2.9 (2.8, 3.0)	3.4 (3.3, 3.5)	3.8 (3.7, 3.9)	4.7 (4.6, 4.8)	< 0.0001
Education (≥ College graduate, %)	61.3	67.4	65.0	64.0	0.35
Household income (≥ 10,000 Yuan, %)	32.0	35.9	34.8	33.6	0.55
Smoking status (%)					
Smoker	2.3	1.4	1.2	1.3	< 0.01
Ex-smoker	0.8	0.6	0.7	0.7	0.94
Non-smoker	96.9	98.1	98.1	98.0	0.02
Drinker (%)					
Everyday	0.4	0.6	0.8	1.0	< 0.01
Sometime	42.3	40.0	39.5	37.4	< 0.001
Ex-drinker	9.7	10.0	9.2	10.0	0.88
Non-drinker	47.6	49.4	50.6	51.6	< 0.01
Employment status (%)					
Managers	36.1	41.4	45.0	41.6	< 0.0001
Professionals	12.5	13.6	11.9	13.2	0.89
Other	51.4	45.0	43.1	45.2	< 0.0001
Family history of diseases (%)					
CVD	30.7	29.2	29.3	27.5	< 0.01
Hypertension	50.6	49.4	49.8	48.0	0.06
Diabetes	26.6	26.7	26.2	22.4	< 0.0001

a *NAFLD* Non-alcoholic fatty liver disease, *CVD* Cardiovascular disease, *BMI* Body mass index, *ALT* Alanine aminotransferase, *AST* Aspartate aminotransferase *DHA* Docosahexaenoic acid, *EPA* Eicosapentaenoic acid

b Analysis of variance or logistic regression

c Least square mean (95% confidence interval) (all such values)

### DF consumption and NAFLD

Table 2 presents the associations between DF consumption and newly diagnosed NAFLD. After multivariable adjustments, higher consumption of DF was associated with lower prevalence of newly diagnosed NAFLD (*P* for trend < 0.01). Compared with the participants in the lowest quartile, the ORs (95% CIs) across increasing consumption of DF were 0.94 (0.82, 1.07), 0.82 (0.70, 0.95), and 0.81 (0.67, 0.97). The same negative association between DF intake and NAFLD was observed in men (*P* for trend = 0.01). The ORs (95% CIs) across increasing consumption of DF were 1 (reference), 0.93 (0.79, 1.01), 0.78 (0.65, 0.94), and 0.78 (0.62, 0.99). However, DF consumption was not significantly associated with newly diagnosed NAFLD in women.

**Table 2:** Associations between total dietary fibre intake and NAFLD by sex ^a^

	Categories of dietary fibre intake				*P* for trend ^b^
All participants (*n* = 23,529)	Level 1	Level 2	Level 3	Level 4	
No. of participants	5883	5882	5881	5883	
No. of participants with NAFLD	1141	1087	1096	1102	
Crude model	Ref	0.94 (0.86, 1.03) ^b^	0.95 (0.87, 1.04)	0.96 (0.87, 1.05)	0.42
Adjusted model 1 ^d^	Ref	0.97 (0.87, 1.09)	0.89 (0.79, 1.00)	0.91 (0.81, 1.02)	0.04
Adjusted model 2 ^e^	Ref	0.94 (0.82, 1.07)	0.82 (0.70, 0.95)	0.81 (0.67, 0.97)	< 0.01
Men (*n* = 10,998)	Level 1	Level 2	Level 3	Level 4	
Intake of dietary fibre (range, g/d)	0.839, 17.417	17.420, 24.134	24.135, 33.692	33.697, 173.414	
No. of participants	2750	2749	2749	2750	
No. of participants with NAFLD	828	779	770	783	
Crude model	Ref	0.92 (0.82, 1.03)	0.90 (0.80, 1.02)	0.92 (0.82, 1.04)	0.24
Adjusted model 1 ^d^	Ref	0.96 (0.84, 1.01)	0.83 (0.72, 0.96)	0.86 (0.75, 0.99)	0.01
Adjusted model 2 ^e^	Ref	0.93 (0.79, 1.01)	0.78 (0.65, 0.94)	0.78 (0.62, 0.99)	0.01
Women (*n* = 12,531)	Level 1	Level 2	Level 3	Level 4	
Intake of dietary fibre (range, g/d)	0.400, 16.161	16.164, 22.284	22.287, 30.760	30.764, 193.433	
No. of participants	3133	3133	3132	3133	
No. of participants with NAFLD	313	308	326	319	
Crude model	Ref	0.98 (0.83, 1.16)	1.05 (0.89, 1.23)	1.02 (0.87, 1.20)	0.65
Adjusted model 1 ^d^	Ref	0.95 (0.77, 1.16)	0.95 (0.77, 1.16)	0.96 (0.79, 1.17)	0.76
Adjusted model 2 ^e^	Ref	0.94 (0.74, 1.19)	0.88 (0.68, 1.15)	0.85 (0.62, 1.17)	0.32

^a^ *NAFLD* Non-alcoholic fatty liver disease, *CVD* Cardiovascular disease, *BMI* Body mass index, *DHA* Docosahexaenoic acid, *EPA* Eicosapentaenoic acid

^b^ Multiple logistic regression analysis

^c^ Odds ratios (95% confidence interval) (all such values)

^d^ Adjusted for age, sex (only for all participants), and BMI

^e^ Adjusted for age, sex (only for all participants), BMI, type 2 diabetes, hypertension, hyperlipidaemia, physical activity, educational level, household income, smoking status, drinking status, employment status, energy intake (kcal/d), total carbohydrate intake (g/d), total fat intake (g/d), sweet foods intake (g/d), red meat intake (g/d), white meat intake (g/d), DHA + EPA intake (g/d), and family history of CVD, hypertension, and diabetes

### Soluble and insoluble DF consumption and NAFLD

Table 3 presents the associations between soluble and insoluble DF intake and newly-diagnosed NAFLD. Insoluble DF intake was negatively associated with the prevalence of newly-diagnosed NAFLD in the overall population (*P* for trend < 0.001) and men (*P* for trend < 0.0001), but not women (*P* for trend = 0.16) after multivariable adjustments. Compared with the participants in the lowest quartile, the ORs (95% CIs) for the participants in the highest quartile were 0.70 (0.58, 0.85) and 0.60 (0.47, 0.76) in the overall population and men, respectively. No significant association was observed between soluble DF intake and NAFLD.

**Table 3:** Associations between different kinds of dietary fibre intake and NAFLD by sex ^a^

	Categories of dietary fibre intake				*P* for trend ^b^
All participants (*n* = 23,529)	Level 1	Level 2	Level 3	Level 4	
Soluble dietary fibre					
Adjusted model ^d^	Ref	1.00 (0.88, 1.14) ^c^	0.90 (0.78, 1.03)	0.91 (0.77, 1.08)	0.13
Insoluble dietary fibre					
Adjusted model ^d^	Ref	0.91 (0.79, 1.04)	0.86 (0.74, 1.01)	0.70 (0.58, 0.85)	< 0.001
Men (*n* = 10,998)	Level 1	Level 2	Level 3	Level 4	
Soluble dietary fibre (range, g/d)	0.318, 7.393	7.394, 10.838	10.839, 16.636	16.637, 86.605	
Adjusted model ^d^	Ref	1.00 (0.85, 1.17)	0.85 (0.72, 1.02)	0.95 (0.78, 1.16)	0.54
Insoluble dietary fibre (range, g/d)	0.839, 9.382	9.384, 12.561	12.563, 16.841	16.842, 88.567	
Adjusted model ^d^	Ref	0.86 (0.73, 1.02)	0.80 (0.67, 0.97)	0.60 (0.47, 0.76)	< 0.0001
Women (*n* = 12,531)	Level 1	Level 2	Level 3	Level 4	
Soluble dietary fibre (range, g/d)	0.143, 6.702	6.703, 9.874	9.875, 14.948	14.951, 93.307	
Adjusted model ^d^	Ref	0.98 (0.78, 1.23)	0.97 (0.76, 1.23)	0.83 (0.62, 1.11)	0.16
Insoluble dietary fibre (range, g/d)	0.257, 8.762	8.765, 11.798	11.799, 15.689	15.691, 99.987	
Adjusted model ^d^	Ref	1.00 (0.79, 1.27)	0.98 (0.75, 1.29)	0.95 (0.68, 1.32)	0.72

^a^ *NAFLD* Non-alcoholic fatty liver disease, *CVD* Cardiovascular disease, *BMI* Body mass index, *DHA* Docosahexaenoic acid, *EPA* Eicosapentaenoic acid

^b^ Multiple logistic regression analysis

^c^ Odds ratios (95% confidence interval) (all such values)

^d^ Adjusted for age, sex (only for all participants), BMI, type 2 diabetes, hypertension, hyperlipidaemia, physical activity, educational level, household income, smoking status, drinking status, employment status, energy intake (kcal/d), total carbohydrate intake (g/d), total fat intake (g/d), sweet foods intake (g/d), red meat intake (g/d), white meat intake (g/d), DHA + EPA intake (g/d), and family history of CVD, hypertension, and diabetes

### Different sources of DF consumption and NAFLD

**Fig. 1:**
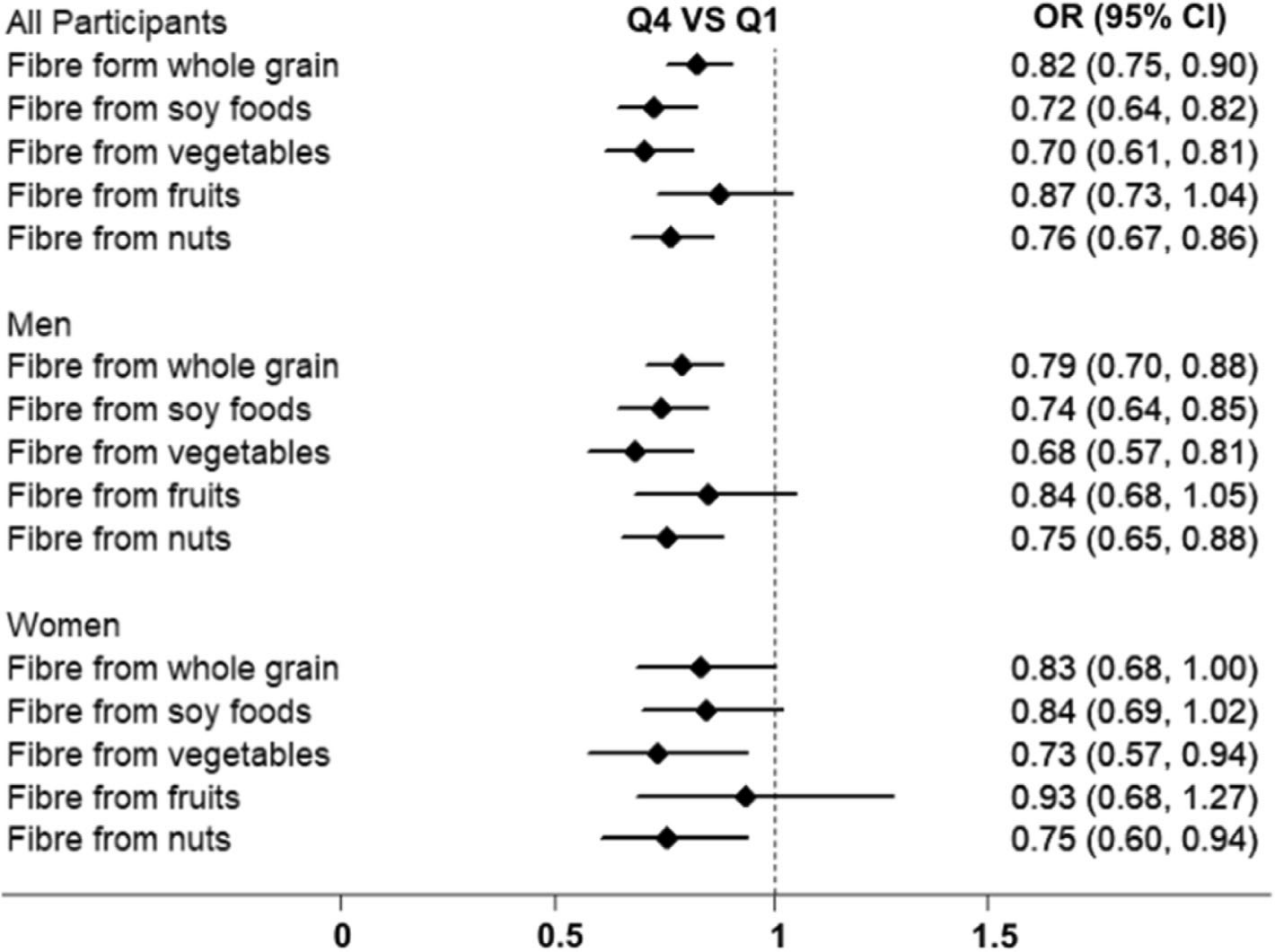
Associations between different sources of dietary fibre intake and NAFLD by sex Adjusted for age, sex (only for all participants), BMI, type 2 diabetes, hypertension, hyperlipidaemia, physical activity, educational level, household income, smoking status, drinking status, employment status, energy intake (kcal/d), total carbohydrate intake (g/d), total fat intake (g/d), sweet foods intake (g/d), red meat intake (g/d), white meat intake (g/d), DHA + EPA intake (g/d), and family history of CVD, hypertension, and diabetes.

Figure 1 presents the associations between different sources of DF intake and the prevalence of newly diagnosed NAFLD. In all participants, compared with the participants in the lowest quartile, the ORs (95% CIs) for the participants in the highest quartile of DF intake from whole grain, soy foods, vegetables, and nuts were 0.82 (0.75, 0.90), 0.72 (0.64, 0.82), 0.70 (0.61, 0.81), and 0.76 (0.67, 0.86) after multivariable adjustments. In men, compared with the participants in the lowest quartile, the ORs (95% CIs) for the participants in the highest quartile of DF intake from whole grain, soy foods, vegetables, and nuts were 0.79 (0.70, 0.88), 0.74 (0.64, 0.85), 0.68 (0.57, 0.81), and 0.75 (0.65, 0.88) after multivariable adjustments. No significant association was observed between DF from fruits intake and NAFLD in the overall population and men. In women, DF from vegetables (Q4 VS Q1: OR, 0.73; 95% CI, 0.57–0.94) and nuts (Q4 VS Q1: OR, 0.75; 95% CI, 0.60–0.94) intake were negatively associated with the prevalence of newly diagnosed NAFLD after multivariable adjustments. No significant association was observed between other sources of DF intake and NAFLD in women.

### Sensitivity analyses

We further excluded participants who had a history of type 2 diabetes or hyperlipidaemia. The associations between DF, soluble DF, and insoluble DF intake and the prevalence of NAFLD in all participants, men, and women were presented in Additional file 1: Table S1. The results between insoluble DF intake and NAFLD were consistent after multivariable adjustments. Compared with the participants in the lowest quartile, the ORs (95% CIs) for participants in the highest quartile of insoluble intake were 0.60 (0.44, 0.80), 0.48 (0.33, 0.70), and 0.83 (0.51, 1.35) in the overall population, men, and women, respectively. However, no significant association was observed between soluble or total DF intake and newly diagnosed NAFLD.

## Discussion

The present study investigated the associations between DF consumption and the prevalence of newly-diagnosed NAFLD in a large general population. The results indicate that DF consumption is associated with lower prevalence of newly-diagnosed NAFLD independent of socio-demographic, behavioural, anthropometric, dietary, and clinical confounding factors in men, but not in women.

Previous clinical-based studies have reported the associations between DF intake and NAFLD [10–13, 22, 23]. Three clinical-based studies (*n* = 50–143) found that DF intake is inversely associated with NASH [10], NAFLD [11], and degree of hepatic steatosis [13]. Yet, another study did not find a difference of DF intake between NAFLD patients and healthy participants in 229 elderly Brazilian (*P* = 0.76) [23]. A case-control study conducted in 36 Chinese participants also suggested that there is no difference in DF intake between NAFLD patients and healthy controls [22]. The mean (standard deviation) intake of DF in cases and control were 21.45 (6.21) and 19.67 (5.47) grams per day [22]. Another study which included 140 Iranians explored the associations between DF intake and NAFLD with adjustments of energy intake, age, and sex [12]. The results suggested that daily DF intake are different in NAFLD patients (mean, 31.2 g; standard deviation, 18.9 g) and healthy controls (mean, 35.3 g; standard deviation, 17.2 g) (*P* = 0.04) [12]. Yet, there are other important confounding factors, such as BMI [24], physical activity [25], and consumption of other nutrients [26], which could affect the associations between DF intake and NAFLD. However, the results from these clinical-based studies were limited by unadjusted statistical analyses, small sample size and clinical-based design. A recent cross-sectional study on the associations between dietary macronutrient composition and the prevalence of NAFLD in a large population with well-adjusted statistical models [14] demonstrated that there is no consistent association between DF intake and the prevalence of NAFLD [14]. However, this study was conducted in an aging population which could also limit the generalisability of their findings. Moreover, due to the nature of the cross-sectional study design, the reverse causation (i.e. participants with NAFLD changed their diet) would affect the associations between DF intake and the prevalence of NAFLD.

Consistent with the findings of previous studies, our findings suggest that higher DF intake, especially insoluble DF, is associated with lower prevalence of newly-diagnosed NAFLD in men even though previous studies may have potential inaccurate results due to small sample size and uncompleted adjustment models [10–13]. A previous study suggested that the associations between dietary factors and NAFLD may be mediated by BMI [14]. However, in the current study, DF intake was still significantly associated with the prevalence of newly diagnosed NAFLD after adjusting for confounders including BMI. Moreover, in the subgroup analysis, we found that insoluble DF, but not soluble DF, contributed to the negative associations between DF intake and the prevalence of NAFLD. There are two plausible explanations for this finding. First, the mechanism that to explain the negative association between insoluble DF intake and NAFLD could be related to SCFAs (short-chain fatty acids). Insoluble DF could be fermented by gut microbe and generate SCFAs, such as butyrate. SCFAs plays an important role in the process of gluconeogenesis which is involved in the development of NAFLD [27]. Meanwhile, SCFAs may inhibit the development of NASH at the epigenetic level as inhibitors of histone deacetylases [27]. A previous study also demonstrated that butyrate may suppress inflammation and thus be associated with a lower prevalence of NAFLD [28]. Furthermore, butyrate could act on the gut-brain neural circuit to improve energy metabolism through enhancing fat oxidation by activating brown adipose tissue [29]. Brown adipose tissue has been found associated with a lower likelihood of NAFLD independently [30]. Second, most of the DF in vegetables and whole grain are insoluble DF, but not soluble DF. Meanwhile, previous studies found that consumption of vegetables and grains favorably affected NAFLD [26, 31].

Moreover, the results between different sources of DF intake and NAFLD suggested that DF intake from whole grain, soy foods, vegetables, and nuts, but not fruits, were negative associated with the prevalence of NAFLD in all participants and men. The non-significant association between fruits intake and NAFLD could be explained by the fructose content in fruits. Fructose has been established as a major risk factor for NAFLD [32]. In the liver, fructose bypasses that whole machinery because it does need phosphofructokinase. Furthermore, most of the fructose that is consumed gets converted to fat [33]. Thus, the content of fructose may in fruits cover up the associations between DF and NAFLD.

However, we did not find a significant association between DF intake and the prevalence of newly diagnosed NAFLD in women consistent with the findings of a previous study that DF intake was significantly correlated with perceived general health status and immune functioning in men, but not women [34]. The underlying mechanisms for sex difference in the associations between DF intake and NAFLD remain uncertain. However, as shown in Fig. 1, the associations between different sources of DF intake and NAFLD tended to be similar in men to those in women. Thus, the null significant associations might be due to smaller number of NAFLD cases in women than in men. Further studies are needed to explore the differences in the associations between DF intake and NAFLD.

The present study has several strengths. First, the analysis on the associations between DF intake and the prevalence of newly diagnosed NAFLD was adjusted for as many confounding factors, including sociodemographic, behavioural, anthropometric, dietary, and clinical confounding factors, as possible. Second, we excluded participants who were clinically diagnosed with NAFLD or those with self-reported NAFLD. Thus, the participants with NAFLD enrolled in the present study were not aware of having NAFLD when filling in the FFQ, and the reverse causation has been minimized as much as possible. Third, the large sample size (23,529) provided sufficient statistical power to detect the associations between DF intake and the prevalence of newly diagnosed NAFLD. Fourth, we further explored the associations between different kinds of DF and DF sources and the prevalence of newly diagnosed NAFLD. The results suggest that insoluble DF and DF from gains, soy foods, vegetables, and nuts were better sources of DF intake. Fifth, we did not collect the information about the amount per once drinking which could be a confounding factor.

Nevertheless, this study also has some limitations. First, there was recall bias, and the food intake reported may be inaccurate due to the nature of the self-report questionnaire. Second, it is impossible to infer causality due to the cross-sectional study design. Third, even though many covariates have been considered, we cannot rule out the possibility that residual and unmeasured factors might contribute to the association observed. Fourth, due to the apparently healthy study population, we used hepatic ultrasonography to detect NAFLD instead of liver biopsy which is the gold standard in the diagnosis of NAFLD. However, hepatic ultrasonography has a sensitivity of 89% and a specificity of 93% in detecting NAFLD and is widely used in population-based studies because of its noninvasiveness and easy accessibility [35].

## Conclusion

A higher intake of insoluble DF is associated with lower prevalence of newly diagnosed NAFLD in men. Moreover, intake DF from whole grain, soy foods, vegetables, and nuts, but not fruits have favorable effect on the prevalence of newly diagnosed NAFLD. Further cohort studies and randomized controlled trials are needed to clarify this finding.

## Supplementary information

**Additional file 1: Table S1.** Associations between dietary fibre intake and NAFLD in participants without type 2 diabetes or hyperlipidaemia by sex ^a^.

## Data Availability

The datasets generated and/or analysed during the current study are not publicly available due to that it is an ongoing cohort study but are available from the corresponding author on reasonable request.

## References

[1] Younossi Z, Tacke F, Arrese M, Sharma BC, Mostafa I, Bugianesi E, et al. Global perspectives on non-alcoholic fatty liver disease and non-alcoholic Steatohepatitis. Hepatol. 2018. 10.1002/hep.30251 30179269

[2] Younossi ZM. Non-alcoholic fatty liver disease - a global public health perspective. J Hepatol. 2018. 10.1016/j.jhep.2018.10.033 30414863

[3] Younossi ZM, Koenig AB, Abdelatif D, Fazel Y, Henry L, Wymer M (2016). Global epidemiology of nonalcoholic fatty liver disease-meta-analytic assessment of prevalence, incidence, and outcomes. Hepatol.

[4] Jia Q, Xia Y, Zhang Q, Wu H, Du H, Liu L (2015). Dietary patterns are associated with prevalence of fatty liver disease in adults. Eur J Clin Nutr.

[5] Leoni S, Tovoli F, Napoli L, Serio I, Ferri S, Bolondi L (2018). Current guidelines for the management of non-alcoholic fatty liver disease: a systematic review with comparative analysis. World J Gastroenterol.

[6] Adriano LS, Sampaio HA, Arruda SP, Portela CL, de Melo MLP, Carioca AA (2016). Healthy dietary pattern is inversely associated with non-alcoholic fatty liver disease in elderly. Br J Nutr.

[7] Fakhoury-Sayegh Nicole, Younes Hassan, Heraoui Gessica, Sayegh Raymond (2017). Nutritional Profile and Dietary Patterns of Lebanese Non-Alcoholic Fatty Liver Disease Patients: A Case-Control Study. Nutrients.

[8] Nguyen V, George J (2015). Nonalcoholic fatty liver disease management: dietary and lifestyle modifications. Semin Liver Dis.

[9] Alba K, Macnaughtan W, Laws AP, Foster TJ, Campbell GM, Kontogiorgos V (2018). Fractionation and characterisation of dietary fibre from blackcurrant pomace. Food Hydrocoll.

[10] Musso G, Gambino R, De Michieli F, Cassader M, Rizzetto M, Durazzo M (2003). Dietary habits and their relations to insulin resistance and postprandial lipemia in nonalcoholic steatohepatitis. Hepatology.

[11] Wehmeyer MH, Zyriax BC, Jagemann B, Roth E, Windler E (2016). Schulze Zur Wiesch J, et al: nonalcoholic fatty liver disease is associated with excessive calorie intake rather than a distinctive dietary pattern. Med (Baltimore).

[12] Zolfaghari H, Askari G, Siassi F, Feizi A, Sotoudeh G (2016). Intake of nutrients, Fiber, and sugar in patients with nonalcoholic fatty liver disease in comparison to healthy individuals. Int J Prev Med.

[13] Papandreou D, Karabouta Z, Pantoleon A, Rousso I (2012). Investigation of anthropometric, biochemical and dietary parameters of obese children with and without non-alcoholic fatty liver disease. Appetite.

[14] Alferink LJ, Kiefte-de Jong JC, Erler NS, Veldt BJ, Schoufour JD, de Knegt RJ, et al. Association of dietary macronutrient composition and non-alcoholic fatty liver disease in an ageing population: the Rotterdam study. Gut. 2018. 10.1136/gutjnl-2017-315940 30064987

[15] Xia Y, Xiang Q, Gu Y, Jia S, Zhang Q, Liu L (2018). A dietary pattern rich in animal organ, seafood and processed meat products is associated with newly diagnosed hyperuricaemia in Chinese adults: a propensity score-matched case-control study. Br J Nutr.

[16] Yang YX, Wang GY, Pan XC, et al. China food composition. 2nd ed. Beijing: Peking University Medical Press; 2009.

[17] Fan JG, Jia JD, Li YM, Wang BY, Lu LG, Shi JP (2011). Guidelines for the diagnosis and management of nonalcoholic fatty liver disease: update 2010: (published in Chinese on Chinese journal of Hepatology 2010; 18:163-166). J Dig Dis.

[18] Craig CL, Marshall AL, Sjostrom M, Bauman AE, Booth ML, Ainsworth BE (2003). International physical activity questionnaire: 12-country reliability and validity. Med Sci Sports Exerc.

[19] James PA, Oparil S, Carter BL, Cushman WC, Dennison-Himmelfarb C, Handler J (2014). 2014 evidence-based guideline for the management of high blood pressure in adults: report from the panel members appointed to the eighth joint National Committee (JNC 8). JAMA.

[20] American Diabetes A (2014). Diagnosis and classification of diabetes mellitus. Diabetes Care.

[21] Joint committee for guideline r: 2016 Chinese guidelines for the management of dyslipidemia in adults**.** J Geriatr Cardiol 2018; 15**:**1–29. 10.11909/j.issn.1671-5411.2018.01.011 PMC5803534 29434622

[22] Cheng Yipeng, Zhang Kewei, Chen Yang, Li Yanchuan, Li Yuzheng, Fu Kuang, Feng Rennan (2016). Associations between Dietary Nutrient Intakes and Hepatic Lipid Contents in NAFLD Patients Quantified by 1H-MRS and Dual-Echo MRI. Nutrients.

[23] de Melo Portela CL, de Carvalho Sampaio HA (2015). Pereira de Melo ML, Ferreira Carioca AA, Maia Pinto FJ, Machado Arruda SP: nutritional status, diet and non-alcoholic fatty liver disease in elders. Nutr Hosp.

[24] VanWagner LB, Khan SS, Ning H, Siddique J, Lewis CE, Carr JJ (2018). Body mass index trajectories in young adulthood predict non-alcoholic fatty liver disease in middle age: the CARDIA cohort study. Liver Int.

[25] Ryu S, Chang Y, Jung HS, Yun KE, Kwon MJ, Choi Y (2015). Relationship of sitting time and physical activity with non-alcoholic fatty liver disease. J Hepatol.

[26] Han JM, Jo AN, Lee SM, Bae HS, Jun DW, Cho YK (2014). Associations between intakes of individual nutrients or whole food groups and non-alcoholic fatty liver disease among Korean adults. J Gastroenterol Hepatol.

[27] Chu H, Duan Y, Yang L, Schnabl B (2019). Small metabolites, possible big changes: a microbiota-centered view of non-alcoholic fatty liver disease. Gut.

[28] Leung C, Rivera L, Furness JB, Angus PW (2016). The role of the gut microbiota in NAFLD. Nat Rev Gastroenterol Hepatol.

[29] Li Z, Yi CX, Katiraei S, Kooijman S, Zhou E, Chung CK (2018). Butyrate reduces appetite and activates brown adipose tissue via the gut-brain neural circuit. Gut.

[30] Yilmaz Y, Ones T, Purnak T, Ozguven S, Kurt R, Atug O (2011). Association between the presence of brown adipose tissue and non-alcoholic fatty liver disease in adult humans. Aliment Pharmacol Ther.

[31] Georgoulis M, Kontogianni MD, Tileli N, Margariti A, Fragopoulou E, Tiniakos D (2014). The impact of cereal grain consumption on the development and severity of non-alcoholic fatty liver disease. Eur J Nutr.

[32] Jensen T, Abdelmalek MF, Sullivan S, Nadeau KJ, Green M, Roncal C (2018). Fructose and sugar: a major mediator of non-alcoholic fatty liver disease. J Hepatol.

[33] Das UN (2015). Sucrose, fructose, glucose, and their link to metabolic syndrome and cancer. Nutrition.

[34] Fernstrand AM, Bury D, Garssen J, Verster JC (2017). Dietary intake of fibers: differential effects in men and women on perceived general health and immune functioning. Food Nutr Res.

[35] Saadeh S, Younossi ZM, Remer EM, Gramlich T, Ong JP, Hurley M (2002). The utility of radiological imaging in nonalcoholic fatty liver disease. Gastroenterology.

