# Supplementary material for: Insoluble dietary fibre intake is associated with lower prevalence of newly-diagnosed non-alcoholic fatty liver disease in Chinese men: a large population-based cross-sectional study
Source: Nutr Metab (Lond). 2020 Jan 13;17:4. doi: 10.1186/s12986-019-0420-1 (PMC6958720; doi:10.1186/s12986-019-0420-1)
Supplement: Supplementary file 1 — Additional file 1: Table S1. Associations between dietary fibre intake and NAFLD in participants without type 2 diabetes or hyperlipidaemia by sex a. [file 12986_2019_420_MOESM1_ESM.docx]

| Additional file 1: Table S1. Associations between total dietary fibre intake and NAFLD in participants without type 2 diabetes or hyperlipidaemia by sex ^a^. | | | | | |
| --- | --- | --- | --- | --- | --- |
|  | Categories of dietary fibre intake | | | | *P* for trend ^b^ |
| **All participants (n = 14,178)** | Level 1 | Level 2 | Level 3 | Level 4 |  |
| Total fibre ^d^ | Ref | 1.03 (0.83, 1.28) | 0.88 (0.70, 1.12) | 0.87 (0.64, 1.16) | 0.18 |
| Soluble dietary fibre ^d^ | Ref | 1.10 (0.90, 1.36) | 1.02 (0.82, 1.28) | 1.05 (0.81, 1.37) | 0.92 |
| Insoluble dietary fibre ^d^ | Ref | 0.77 (0.62, 0.95) | 0.81 (0.64, 1.03) | 0.60 (0.44, 0.80) | <0.01 |
| **Men (n = 5,752)** | Level 1 | Level 2 | Level 3 | Level 4 |  |
| Total fibre ^d^ | Ref | 1.04 (0.80, 1.35) | 0.79 (0.59, 1.06) | 0.87 (0.60, 1.26) | 0.22 |
| Soluble dietary fibre ^d^ | Ref | 1.17 (0.90, 1.50) | 0.95 (0.72, 1.26) | 1.18 (0.85, 1.63) | 0.57 |
| Insoluble dietary fibre ^d^ | Ref | 0.80 (0.62, 1.04) | 0.76 (0.57, 1.03) | 0.48 (0.33, 0.70) | <0.001 |
| **Women (n = 8,426)** | Level 1 | Level 2 | Level 3 | Level 4 |  |
| Total fibre ^d^ | Ref | 1.01 (0.70, 1.47) | 1.18 (0.79, 1.77) | 0.86 (0.53, 1.41) | 0.57 |
| Soluble dietary fibre ^d^ | Ref | 1.04 (0.73, 1.48) | 1.25 (0.86, 1.81) | 0.92 (0.59, 1.44) | 0.64 |
| Insoluble dietary fibre ^d^ | Ref | 0.73 (0.51, 1.06) | 0.92 (0.61, 1.39) | 0.83 (0.51, 1.35) | 0.82 |

^a^ NAFLD, non-alcoholic fatty liver disease; CVD, cardiovascular disease; BMI, body mass index; DHA, docosahexaenoic acid; EPA, eicosapentaenoic acid.

^b^ Multiple logistic regression analysis.

^c^ Odds ratios (95% confidence interval) (all such values).

^d^ Adjusted for age, sex (only for all participants), BMI, hypertension, physical activity, educational level, household income, smoking status, drinking status, employment status, energy intake (kcal/d), total carbohydrate intake (g/d), total fat intake (g/d), sweet foods intake (g/d), red meat intake (g/d), white meat intake (g/d), DHA+EPA intake (g/d), and family history of CVD, hypertension, and diabetes.
